# Genetic Subtypes, Pretreatment Drug Resistance, and Associated Factors Among Individuals Newly Diagnosed With HIV-1 in Zhejiang Province From 2022 to 2024: Cross-Sectional Study

**DOI:** 10.2196/96139

**Published:** 2026-09-17

**Authors:** Wei Cheng, Qin Fan, Jiafeng Zhang, Jun Jiang, Jiezhe Yang, Yun Xu, Lin Chen, Chengliang Chai, Xiaohong Pan

**Affiliations:** 1 Zhejiang Provincial Center for Disease Control and Prevention, 3399 Binsheng Road, Binjiang District, Hangzhou, Zhejiang, Hangzhou, 310051, China, 86 057187115191

**Keywords:** genetic subtypes, pretreatment drug resistance, exposure prophylaxis, associated factors, HIV-1

## Abstract

**Background:**

Pretreatment drug resistance (PDR) poses an increasing threat to the effectiveness of antiretroviral therapy and treatment-as-prevention strategies. Zhejiang Province, an economically developed region in eastern China with high population mobility, experiences complex HIV-1 subtype dynamics and evolving resistance patterns. However, recent population-based evidence integrating HIV genetic subtypes, PDR prevalence, and associated factors remains limited.

**Objective:**

This study aimed to characterize HIV-1 subtype distribution, estimate the prevalence of PDR, and identify associated demographic, virological, and prophylaxis-related factors among newly diagnosed individuals in Zhejiang Province from 2022 to 2024.

**Methods:**

We conducted a cross-sectional study among adults newly diagnosed with HIV-1 in Zhejiang Province between 2022 and 2024. Demographic, epidemiological, laboratory, and exposure prophylaxis information was collected through the provincial HIV surveillance system. Partial Pol gene sequences were amplified and sequenced to determine HIV subtypes and drug resistance mutations. PDR was interpreted using the Stanford University HIV Drug Resistance Database. Logistic regression models were applied to identify factors associated with PDR.

**Results:**

Among 8798 newly diagnosed individuals, the predominant subtypes were CRF07_BC (n=3913, 44.5%), CRF01_AE (n=2524, 28.7%), and CRF08_BC (n=954, 10.8%), with significant heterogeneity across age groups, sexes, transmission routes, and diagnosis years (*P*<.001). Overall PDR prevalence was 5.7% (n=504), with resistance mainly to nonnucleoside reverse-transcriptase inhibitors (n=352, 4.0%). The most frequently detected mutations were K103N, M184V, and M46L. Multivariable analysis showed that higher baseline CD4 cell counts, diagnosis in 2023 and 2024, and infection with the CRF01_AE subtype were independently associated with increased odds of PDR. Pre-exposure prophylaxis was not associated with overall PDR; however, drug-specific analyses demonstrated significant associations between exposure prophylaxis and pretreatment resistance to emtricitabine (*P*<.001) and lamivudine (*P*<.001).

**Conclusions:**

HIV-1 genetic diversity in Zhejiang Province remains complex and dynamic, accompanied by an increasing prevalence of PDR. Although exposure prophylaxis was not linked to overall PDR, its association with emtricitabine-specific resistance highlighted the need for strengthened HIV-1 testing prior to prophylaxis initiation and enhanced resistance surveillance, but they require confirmation in future studies.

## Introduction

Despite substantial progress in antiretroviral (ARV) therapy (ART) coverage, HIV transmission and AIDS-related mortality remain unacceptably high. According to the Joint United Nations Programme on HIV/AIDS, an estimated 1.3 million new HIV infections and 630,000 AIDS-related deaths occurred worldwide in 2024, far exceeding the global targets set for 2025 [1]. The epidemic continues to disproportionately affect low- and middle-income countries and key populations [1,2]. Consequently, HIV infection remains a major public health concern requiring sustained and adaptive interventions [1,2].

The concept of “treatment as prevention” has become a cornerstone of global HIV control strategies. Early initiation of ART effectively suppresses viral replication, reduces onward transmission, and significantly improves survival and quality of life among people living with HIV [2]. Nevertheless, the effectiveness of treatment as prevention is increasingly threatened by the growing prevalence of pretreatment drug resistance (PDR) [3,4]. PDR refers to any drug-resistant virus detected in ARV drug–naive individuals initiating ART or individuals with previous ARV drug exposure initiating or reinitiating first-line ART [5]. PDR can compromise first-line ART regimens, increase the risk of virological failure, and impose substantial economic burdens on health care systems by necessitating the use of more expensive second-line therapies [3,4,6]. Therefore, continuous monitoring of PDR and identification of its associated factors are essential for optimizing treatment strategies and preventing the spread of resistant HIV strains.

In recent years, integrase strand transfer inhibitor (INSTI)–based regimens have become the preferred first-line treatment strategy worldwide [5]. Although the prevalence of INSTI resistance remains relatively low worldwide, the increasing use of INSTIs has been accompanied by the emergence of resistance-associated mutations in both treatment-experienced and, occasionally, treatment-naive populations [2,5,7]. In China, dolutegravir-based regimens have been progressively incorporated into national treatment guidelines; however, population-level surveillance data on INSTI resistance remain limited [8]. Long-acting ARV agents, including capsid inhibitors such as lenacapavir, are expanding the therapeutic landscape. Although resistance to capsid inhibitors remains uncommon, treatment-emergent capsid resistance mutations have been documented [9], highlighting the importance of proactive resistance surveillance as new drug classes are introduced into clinical practice.

Zhejiang Province, located in southeastern China, is an economically developed region characterized by high population mobility. Rapid socioeconomic development and population mobility may facilitate the frequent movement of HIV-positive individuals and ongoing viral recombination, potentially leading to a more complex HIV genotyping distribution and posing challenges to local prevention and control. Several studies have investigated HIV drug resistance in the Zhejiang Province. In Hangzhou, Zhang et al [10] reported a transmitted drug resistance (TDR) prevalence of 4.0% among newly diagnosed individuals in 2013, with CRF01_AE as the dominant subtype. In Ningbo, Hong et al [11] observed a TDR prevalence of 6.1% from 2018 to 2021, with K103N as the most frequent mutation and CRF07_BC as the major genotype. In Wenzhou, Zhang et al [12] reported a TDR prevalence of 5.7% between 2020 and 2023 and identified CRF08_BC as a subtype associated with a higher resistance risk. Recently, in Lishui, Li et al [13] observed a TDR prevalence of 12.1% from 2020 to 2023, with resistance predominantly to nonnucleoside reverse-transcriptase inhibitors (NNRTIs). These studies indicate that HIV-1 genetic diversity and drug resistance are present across Zhejiang but vary by city, period, and study population [10-13]. Therefore, a standardized, population-based study covering the entire province over a recent period is urgently needed to characterize HIV subtype distribution, estimate PDR prevalence using a consistent definition, and identify associated demographic, virological, and prophylaxis-related factors. Moreover, the expanding use of pre-exposure prophylaxis (PrEP) and postexposure prophylaxis (PEP) has raised concerns about the potential selection of drug resistance when these interventions are initiated during undiagnosed HIV infection or when adherence is suboptimal [5]. Nonadherence could allow for breakthrough viremia and the emergence of resistant strains, which might then be transmitted or persist as pretreatment resistance. However, empirical evidence from real-world settings in China remains scarce. Against this background, a systematic analysis integrating viral genetic subtypes, PDR, and associated demographic and behavioral factors is urgently needed.

Therefore, this study aimed to characterize HIV subtype distribution, estimate the prevalence of PDR, and identify associated demographic, virological, and prophylaxis-related factors among newly diagnosed individuals in Zhejiang Province from 2022 to 2024.

## Methods

### Study Participants and Data Collection

This cross‐sectional study was conducted in Zhejiang Province. This province is home to over 65 million local residents spread across 11 metropolitan areas. Inclusion criteria were as follows: (1) new diagnosis with HIV-1 between January 1, 2022, and December 31, 2024; (2) age of 18 years or above; and (3) provision of informed consent for PDR testing and successful provision of blood samples. According to the law, all individuals newly diagnosed with HIV must be reported to the China Notifiable Infectious Disease Reporting Information System. After confirming the HIV diagnosis, staff at local Center for Disease Control and Prevention (CDC) conducted face-to-face interviews with the patients to collect baseline demographic characteristics (sex, age, educational level, and marital status) and epidemiological data (route of infection). Blood samples were obtained from patients who provided informed consent to record their baseline CD4 cell counts before the initiation of ART. All collected information was reported to the Zhejiang Province database of the national data information system for comprehensive HIV/AIDS control. During the study period, in addition to collecting common information, data on exposure prophylaxis history (ie, whether the patient had such a history and, if yes, further classification as PrEP or PEP) were collected through patient self-report. To ensure strict confidentiality of personal data, all blood samples were assigned numeric codes linked to corresponding sociodemographic information. During the study period, the total number of newly diagnosed cases in the province was 10,485, of whom 9668 (92.2%) had blood samples collected. The success rate for amplification and sequencing was 91.0% (8798/9668).

### HIV-1 RNA Extraction, Amplification, and Sequencing

Total viral RNA was extracted from plasma specimens in accordance with the manufacturer’s protocol using a viral RNA extraction kit (Tianlong Science and Technology). A Pol gene fragment (1316 bp, HXB2 coordinates 2147-3462) encoding entire protease and the first 299 residues of reverse transcriptase was amplified via reverse-transcriptase polymerase chain reaction (PCR; RT-PCR) followed by nested PCR. Commercially available amplification kits were used, including the PrimeScript One Step RT-PCR kit version 2 (Takara Bio) and the Ex Taq kit (Takara Bio). Primer sequences and thermal cycling conditions were as previously described [10]. Amplification was performed in the laboratories of the 11 city-level CDC. PCR products were analyzed using 1% agarose gel electrophoresis. Target amplicons were subsequently purified and sequenced by Tsingke Biotech using a 3730XL DNA sequencer (Applied Biosystems) with 5 overlapping primers.

### HIV Genotyping and Drug Resistance Testing

Sequence trimming, contig assembly, and interpretation of ambiguous bases were conducted using Sequencher (version 5.4.6; Gene Codes Corporation). In accordance with a previous study [14], ambiguous bases were considered if the signal intensity of the secondary peak exceeded 20% of the primary peak, whereas sequences containing 5% ambiguous bases or more were excluded from the study. All sequences were submitted to the HIV-1 sequence quality control tool for quality verification, which included the screening and exclusion of hypermutation. Subsequently, assembled sequences were aligned using ClustalW within BioEdit (version 7.2.0). HIV-1 subtypes were determined using 2 independent methods: the online automated subtyping tool COMET (Context-Based Modeling for Expeditious Typing) and phylogenetic analysis [15]. Phylogenetic analysis was used as the definitive determination when the 2 tools yielded inconsistent results. Phylogenetic trees were constructed in MEGA (version 6.0) using the neighbor-joining method based on the Kimura 2-parameter model, with bootstrap validation of 1000 replicates. Reference sequences encompassing major HIV-1 subtypes and circulating recombinant forms were obtained from the Los Alamos National Laboratory HIV sequence database. Potential intersubtype recombination events were analyzed using the Recombination Identification Program (version 3.0) [16]. Unclassified sequences were considered Unique Recombinant Form, and the recombination pattern was confirmed using a jumping profile hidden Markov model and SimPlot (version 3.5.1) [14].

Resistance interpretation for each ARV drug was performed using the Stanford University HIV Drug Resistance Database (version 9.8), with resistance defined as low level or higher (score of ≥15) [17,18]. The presence of one or more drugs with resistance was deemed to constitute PDR.

### Statistical Analyses

Categorical variables are presented as frequencies and percentages. Between-group comparisons were performed using the Pearson chi-square test. Factors associated with PDR were identified using logistic regression analysis. Variables with a *P* value below .10 in univariate analysis, along with factors previously reported to be associated with PDR, were included in the multivariable model. We applied Firth logistic regression when the events per variable (EPV) were less than 10. All analyses were conducted using the R statistical software (version 4.1.2; R Foundation for Statistical Computing). A 2-sided *P* value below .05 was considered statistically significant in the final model.

### Ethical Considerations

This study was a retrospective analysis of deidentified data from the Zhejiang provincial HIV surveillance system. The ethics committee of the Zhejiang provincial CDC branch approved the use of these data for research purposes (approval number 2025-077-01) and waived the requirement for individual informed consent because the data were anonymized and the study posed minimal risk. This study was conducted in accordance with the principles of the Declaration of Helsinki.

## Results

### Characteristics of Study Participants

A total of 8798 individuals newly diagnosed with HIV in Zhejiang Province from 2022 to 2024 were included. Most (n=7341, 83.4%) were male and aged 50 years or older (n=3274, 37.2%). More than half (n=5173, 58.8%) had educational levels of middle school or lower. Heterosexual transmission accounted for 54.8% (n=4820), whereas homosexual transmission represented 44.3% (n=3900). Over 50% of cases (n=4479, 50.9%) were identified through medical institutions, and 70.5% (n=6202) had CD4 counts below 350 cells/μL at diagnosis. In total, 1.0% (n=91) of individuals had a history of PEP or PrEP use. The predominant HIV subtypes were CRF07_BC (n=3913, 44.5%) and CRF01_AE (n=2524, 28.7%; Table 1).

**Table 1.** Characteristics of individuals with newly diagnosed HIV infection in Zhejiang Province (2022-2024; N=8798).

Variables	Individuals, n (%)
Age group (y)	
18-29	2277 (25.9)
30-39	1713 (19.5)
40-49	1534 (17.4)
≥50	3274 (37.2)
Sex	
Male	7341 (83.4)
Female	1457 (16.6)
Educational level	
Illiterate	517 (5.9)
Primary school	1912 (21.7)
Middle school	2744 (31.2)
High school or technical secondary school	1668 (19.0)
College degree or higher	1957 (22.2)
Marital status	
Unmarried, divorced, or widowed	5100 (58.0)
Married or cohabiting	3633 (41.3)
Other	65 (0.7)
Transmission route	
Heterosexual	4820 (54.8)
Homosexual	3900 (44.3)
Other	78 (0.9)
HIV diagnosis approach	
Medical institution	4479 (50.9)
VCT^a^	1871 (21.3)
STD^b^ clinic	1015 (11.5)
Other	1433 (16.3)
CD4 cell count at diagnosis (cells/μL)	
0-199	3087 (35.1)
200-349	3115 (35.4)
350-499	1590 (18.1)
≥500	861 (9.8)
Missing	145 (1.6)
Confirmation year	
2022	2903 (33.0)
2023	3066 (34.8)
2024	2829 (32.2)
Exposure prophylaxis history	
PEP^c^	46 (0.5)
PrEP^d^	45 (0.5)
None	8707 (99.0)
HIV subtype	
B	168 (1.9)
C	119 (1.4)
CRF01_AE	2524 (28.7)
CRF07_BC	3913 (44.5)
CRF08_BC	954 (10.8)
CRF55_01	304 (3.5)
CRF85_BC	148 (1.7)
URF^e^ (CRF01_AE/CRF07_BC)	334 (3.8)
Other	334 (3.8)

a VCT: voluntary counseling and testing.

b STD: sexually transmitted disease.

c PEP: postexposure prophylaxis.

d PrEP: pre-exposure prophylaxis.

e URF: Unique Recombinant Form.

### HIV Subtype Distribution

**Figure 1.**
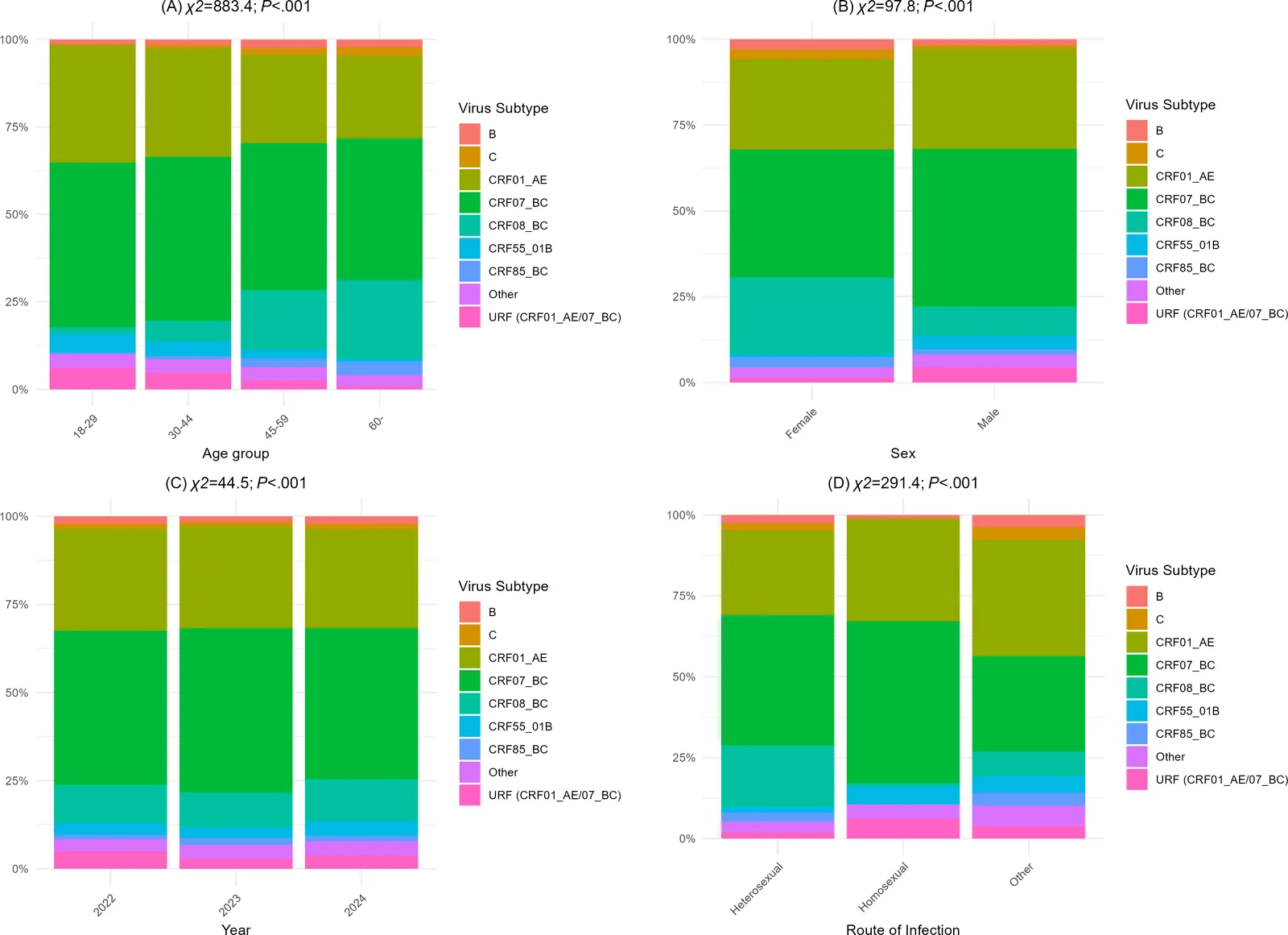
HIV genetic subtype distribution across (A) age groups, (B) sexes, (C) diagnosis years, and (D) transmission routes among newly diagnosed HIV infections in Zhejiang Province (2022-2024). URF: Unique Recombinant Form.

Although CRF07_BC was the dominant circulating subtype, significant differences in subtype distribution were observed across age groups, sexes, routes of transmission, and years of diagnosis (*P*<.001 in all cases). The proportion of CRF07_BC and CRF01_AE decreased with increasing age, whereas the proportion of CRF08_BC increased. Compared to male individuals, female individuals showed a relatively higher proportion of CRF08_BC. Temporal analysis also revealed changes in subtype composition over the 3-year study period. In terms of transmission route, CRF07_BC and CRF01_AE were highly prevalent among homosexual transmissions, whereas CRF08_BC constituted a larger share among heterosexual transmissions (Figure 1).

### Drug Resistance Mutation Patterns

**Figure 2.**
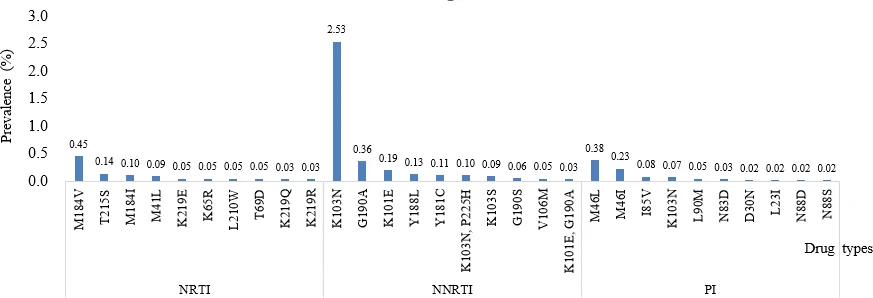
Prevalence of the top 10 detected HIV pretreatment drug resistance mutations in Zhejiang Province (2022-2024). NNRTI: nonnucleoside reverse-transcriptase inhibitor; NRTI: nucleoside reverse-transcriptase inhibitor; PI: protease inhibitor.

Of the 8798 patients involved in our study, 529 (6.0%) had drug resistance mutations. The proportion of participants carrying at least one nucleoside reverse-transcriptase inhibitor (NRTI), NNRTI, and protease inhibitor (PI)–associated mutation was 1.2% (n=113), 3.9% (n=346), and 1.1% (n=92), respectively. The 10 most prevalent drug resistance mutations across NRTIs, NNRTIs, and PIs are shown in Figure 2. Among detected resistance mutations, the most common NRTI mutation was M184V (n=40, 0.5%). The K103N mutation (n=223, 2.5%) predominated among NNRTI-resistant strains, whereas M46L (n=33, 0.4%) was the most frequently observed PI resistance mutation.

### Prevalence of PDR

**Figure 3.**
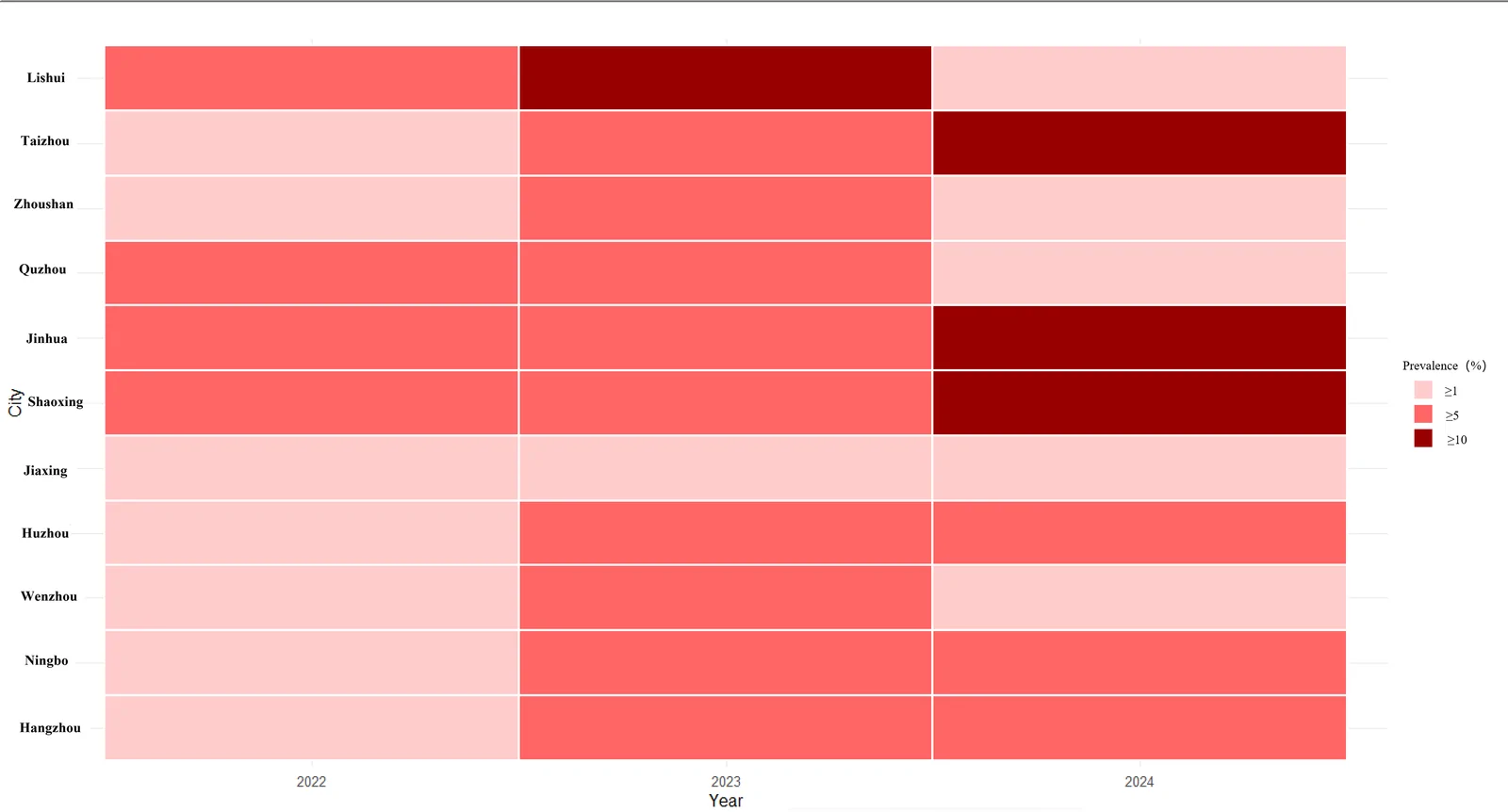
Prevalence of HIV pretreatment drug resistance across 11 metropolitan areas among newly diagnosed HIV infections in Zhejiang Province (2022-2024).

**Figure 4.**
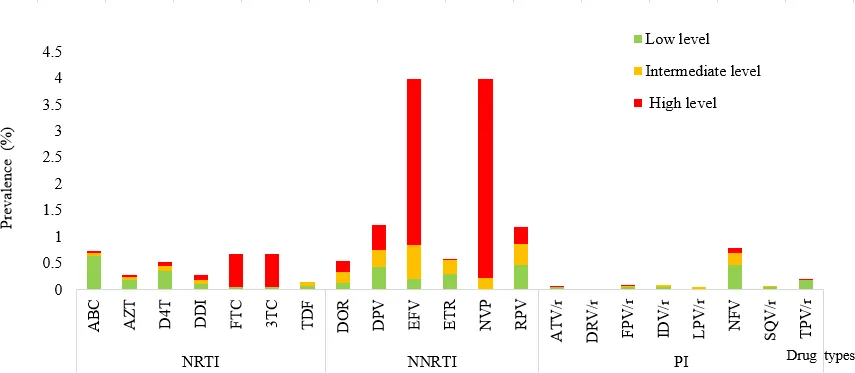
Prevalence and levels of HIV pretreatment drug resistance to different antiretroviral drugs in Zhejiang Province (2022-2024). 3TC: lamivudine; ABC: abacavir; ATV/r: atazanavir/r; AZT: zidovudine; D4T: stavudine; DDI: didanosine; DOR: doravirine; DPV: dapivirine; DRV/r: darunavir/r; EFV: efavirenz; ETR: etravirine; FPV/r: fosamprenavir/r; FTC: emtricitabine; IDV/r: indinavir/r; LPV: lopinavir/r; NFV: nelfinavir; NNRTI: nonnucleoside reverse-transcriptase inhibitor; NRTI: nucleoside reverse-transcriptase inhibitor; NVP: nevirapine; PI: protease inhibitor; RPV: rilpivirine; SQV/r: saquinavir/r; TDF: tenofovir; TPV/r: tipranavir/r.

Overall, 5.7% (504/8798) of the individuals had PDR. The prevalence of PDR to NRTIs, NNRTIs, and PIs was 1.2% (101/8798), 4.0% (352/8798), and 0.9% (82/8798), respectively. PDR prevalence remained relatively low in most regions. However, nearly half of the municipalities exhibited an increasing trend. Meanwhile, areas such as Shaoxing (19/190, 10%), Jinhua (40/377, 10.6%), and Taizhou (25/248, 10.1%) exhibited relatively higher PDR, with a prevalence of more than 10% in 2024 (Figure 3). The degree of resistance to each ARV drug is shown in Figure 4. Abacavir exhibited the highest resistance rate among NRTIs (65/8798, 0.7%), with emtricitabine (FTC) and lamivudine (3TC) both showing rates of 0.6% (60/8798). For NNRTIs, nevirapine (NVP) and efavirenz (EFV) resistance was the most common (351/8798, 4.0%), followed by dapivirine (108/8798, 1.2%). The percentages of high‐level resistance to NVP and EFV were notably high at 3.8% (331/8798) and 3.2% (277/8798), respectively. Among PIs, nelfinavir resistance was the most common (69/8798, 0.8%), followed by tipranavir (18/8798, 0.2%). The PDR prevalence was significantly across different confirmation years and HIV subtypes (Table 2).

**Table 2.** Prevalence of pretreatment drug resistance and associated factor analysis among newly diagnosed HIV infections in Zhejiang Province (2022-2024).

Variables	Individuals, (n=504) (%)	*P* value (univariate analysis)	aOR^a^ (95% CI)	*P* value (multivariate analysis)
Age group (y)		.28		
18-29	124 (5.5)		0.89 (0.65-1.23)	.48
30-39	115 (6.7)		1.18 (0.88-1.57)	.26
40-49	85 (5.5)		0.95 (0.72-1.26)	.74
≥50	180 (5.5)		Reference	—^b^
Sex		.58		
Male	416 (5.7)		Reference	—
Female	88 (6.0)		1.10 (0.84-1.43)	.50
Educational level		.54		
Illiterate	24 (4.6)		Reference	—
Primary school	104 (5.4)		1.19 (0.75-1.88)	.46
Middle school	171 (6.2)		1.37 (0.86-2.17)	.18
High school or technical secondary school	90 (5.4)		1.14 (0.69-1.88)	.61
College degree or higher	115 (5.9)		1.20 (0.72-1.99)	.50
Marital status		.47		
Unmarried, divorced, or widowed	293 (5.8)		Reference	—
Married or cohabiting	205 (5.6)		0.99 (0.80-1.23)	.93
Other	6 (9.2)		1.71 (0.72-4.06)	.23
Transmission route		.70		
Heterosexual	272 (5.6)		Reference	—
Homosexual	229 (5.9)		1.03 (0.82-1.30)	.80
Other	3 (3.9)		0.6 (0.18-1.96)	.40
HIV diagnosis approach		.27		
Medical institution	118 (6.3)		Reference	—
VCT^c^	262 (5.9)		1.04 (0.81-1.32)	.77
STD^d^ clinic	56 (5.5)		0.93 (0.69-1.27)	.65
Other	68 (4.8)		0.77 (0.59-1.02)	.07
CD4 cell count at diagnosis (cells/μL)		.05		
0-199	154 (5.0)		Reference	—
200-349	175 (5.6)		1.20 (0.96-1.51)	.11
350-499	111 (7.0)		1.58 (1.22-2.05)	.001
≥500	57 (6.6)		1.45 (1.05-2.01)	.02
Missing	7 (4.8)		0.98 (0.45-2.16)	.97
Confirmation year		.001		
2022	105 (3.6)		Reference	—
2023	206 (6.7)		1.96 (1.54-2.49)	.001
2024	193 (6.8)		1.97 (1.54-2.52)	.001
Exposure prophylaxis history		.10		
None	496 (5.7)		Reference	—
PEP^e^	2 (4.4)		0.62 (0.15-2.58)	.51
PrEP^f^	6 (13.3)		2.04 (0.84-4.96)	.11
HIV subtype		.001		
CRF07_BC	199 (5.1)		Reference	—
CRF01_AE	192 (7.6)		1.60 (1.30-1.96)	.001
CRF08_BC	53 (5.6)		1.18 (0.85-1.63)	.34
CRF55_01	14 (4.6)		0.92 (0.52-1.60)	.76
Other	46 (4.2)		0.84 (0.60-1.17)	.29

a aOR: adjusted odds ratio.

b Not applicable

c VCT: voluntary counseling and testing.

d STD: sexually transmitted disease.

e PEP: postexposure prophylaxis.

f PrEP: pre-exposure prophylaxis.

### Factors Associated With PDR

Multivariable logistic regression analysis identified several factors independently associated with PDR. Individuals with a baseline CD4 cell count of 350 cells/μL or higher were more likely to have PDR than those with a baseline CD4 cell count of less than 50 cells/μL (350-499 cells/μL: adjusted odds ratio [aOR] 1.58, 95% CI 1.22-2.05; ≥500 cells/μL: aOR 1.45, 95% CI 1.05-2.01). Patients diagnosed in 2023 and 2024 had approximately 2-fold higher odds of having PDR than those diagnosed in 2022 (eg, 2024: aOR 1.97, 95% CI 1.54-2.52). In addition, HIV-1 subtype was significantly associated with PDR. Compared with CRF07_BC, infection with the CRF01_AE subtype was associated with a significantly increased risk of PDR (aOR 1.60, 95% CI 1.30-1.96). No significant associations were observed for age, sex, marital status, transmission route, or HIV diagnosis approach. No statistically significant differences in PDR prevalence were observed between individuals with a history of PrEP or PEP use and those without prior exposure prophylaxis.

### Association Between Exposure Prophylaxis and Drug-Specific Pretreatment Resistance

We examined the association between prior exposure prophylaxis (PrEP and PEP) and resistance to tenofovir (TDF), FTC, and 3TC in the 8798 newly diagnosed individuals. Only 13 events of TDF resistance occurred in the entire study sample. Owing to an EPV value of less than 1, multivariate analysis was not performed, and only unadjusted estimates were presented (odds ratio [OR] 17.76, 95% CI 3.88‐81.31). For FTC and 3TC, the prevalence of pretreatment resistance was 0.6% (55/8707) among those without prophylactic exposure and 5.5% (5/91) among those with prophylactic exposure. Univariate analysis showed a significant association (OR 9.15, 95% CI 3.57‐23.41). After adjusting for age, sex, educational level, transmission route, marital status, HIV diagnosis approach, baseline CD4 cell count, diagnosis year, and HIV subtype using Firth logistic regression, the association remained significant (aOR 7.61, 95% CI 3.01‐19.25; Table 3).

**Table 3.** Association between exposure prophylaxis and pretreatment tenofovir (TDF), emtricitabine (FTC), and lamivudine (3TC) resistance among newly diagnosed HIV infections in Zhejiang Province (2022-2024).

Exposure prophylaxis	PDR^a^ prevalence, n/N (%)	Univariate analysis		Multivariate analysis	
		OR^b^ (95% CI)	*P* value	aOR^c^ (95% CI)	*P* value
TDF					
No	11/8707 (0.1)	Reference	—^d^	Reference	—^d^
Yes	2/91 (2.2)	17.76 (3.88-81.31)	.001	—^e^	—^e^
FTC					
No	55/8707 (0.6)	Reference	—^d^	Reference	—^d^
Yes	5/91 (5.5)	9.15 (3.57-23.41)	.001	7.61 (3.01-19.25)^f^	.001
3TC					
No	55/8707 (0.6)	Reference	—^d^	Reference	—^d^
Yes	5/91 (5.5)	9.15 (3.57-23.41)	.001	7.61 (3.01-19.25)^f^	.001

a PDR: pretreatment drug resistance.

b OR: odds ratio.

c aOR: adjusted odds ratio.

d Not applicable

e Not available because of events per variable <1.

f Adjusting for age, sex, educational level, transmission route, marital status, HIV diagnosis approach, CD4 cell count at diagnosis, confirmation year, and HIV subtype using Firth logistic regression.

## Discussion

This study provided the first representative description of the PDR trend along with HIV-1 variant dynamics over 3 years in Zhejiang Province. This study showed that the most prevalent HIV genotype was CRF07_BC (3913/8798, 44.5%), followed by CRF01_AE (2524/8798, 28.7%) and CRF08_BC (954/8798, 10.8%). Moreover, HIV-1 subtypes showed heterogeneity in distribution across age groups, sexes, transmission routes, and diagnosis years. PDR was prevalent in 5.7% (504/8798) of the study population but presenting an upward trend, with the highest prevalence among NNRTIs (351/8798, 4.0%). In addition, baseline CD4 cell count, diagnosis year, and HIV subtype were independently associated with PDR. Although exposure prophylaxis was not associated with overall PDR, it was significantly associated with pretreatment resistance to FTC and 3TC.

We observed that CRF07_BC and CRF01_AE remained the dominant circulating HIV subtypes, consistent with previous reports from Tianjin, Chongqing, Beijing, Shenzhen, and Xi’an [19-23]. Age-specific differences in subtype composition suggest that distinct transmission dynamics operate across generations. Sex- and transmission route–specific variations in subtype distribution further emphasize the role of social and behavioral structures in shaping HIV epidemics. The statistically significant variation in subtype distribution by diagnosis year suggests that the local HIV epidemic is dynamic rather than static, with ongoing introductions, expansions, or contractions of specific viral lineages. Simultaneously, the complexity and diversity of HIV genotypes may be attributed to population flow and the spread of resistant strains [6]. The observed demographic and temporal heterogeneity in subtype distribution underscores the need to integrate molecular epidemiology into routine surveillance frameworks.

We observed notable geographical heterogeneity in PDR prevalence across Zhejiang Province, with some municipalities (eg, Jinhua and Taizhou) showing PDR prevalence exceeding 10% in 2024 (40/377, 10.6% and 25/248, 10.1%, respectively). This uneven distribution may be explained by the higher rates of CRF01_AE (which is associated with PDR) and clustered transmission of resistant strains [24], which requires further research. PDR has been reported in different regions of China, with a prevalence of 4.8% in Shanghai [25], 23.1% in Shenzhen [19], 7.4% in Guangzhou [17], 10.5% in Chongqing [26], and 18.3% in Xi’an [20]. The varying prevalence of PDR reported in different studies is likely attributed to multifactorial causes, including distinct definition, local differences in HIV subtype distribution, transmission dynamics, and accessibility and quality of ART programs. Therefore, continuous surveillance is essential to detect early warning signals and guide timely policy responses [19].

Notably, higher CD4 cell counts at diagnosis were associated with increased PDR risk. This finding may reflect the predominance of TDR among individuals diagnosed at earlier stages of infection, who are more likely to harbor resistant strains acquired from treated partners and probably no adherence to treatment. In addition, the significantly higher PDR prevalence among individuals diagnosed in 2023 and 2024 suggests an accelerating spread of drug-resistant HIV strains in recent years. These findings underscore the need for comprehensive adherence support interventions, including peer educators, psychological counseling, and ensuring a stable drug supply, to prevent treatment failure and the subsequent spread of resistant strains. We also found that infection with the CRF01_AE subtype was independently associated with a higher risk of PDR compared with CRF07_BC. This observation aligns with previous studies reporting a higher burden of resistance mutations among CRF01_AE strains in other areas [20,27], which supports enhanced resistance surveillance in populations where CRF01_AE is prevalent as it may facilitate a better understanding of local resistance dynamics.

While prior PrEP or PEP use was not associated with overall PDR, drug-specific analyses revealed associations with pretreatment resistance to TDF, FTC, and 3TC—the backbone agents of most prophylaxis regimens. This selective pattern is biologically plausible and warrants careful interpretation. Initiation of PrEP or PEP during undiagnosed acute infection may expose replicating viruses to dual-NRTI pressure, facilitating selection of M184V-, M184I-, or K65R-related resistance pathways [28,29]. In addition, partial adherence to PrEP but not complete nonadherence may favor resistant variant emergence [28]. Alternatively, prophylaxis users may belong to higher-incidence sexual networks where resistant strains are more likely to circulate, reflecting confounding by transmission intensity rather than direct selection pressure. The magnitude of the association observed for TDF resistance was substantial, but the wide CI and inability to adjust for confounders due to the low event count (EPV<1) need to be confirmed in future studies.

Crucially, the potential emergence of drug-specific resistance must be weighed against the substantial epidemiological benefits of PrEP and PEP scale-up. Mathematical modeling and real-world implementation data consistently indicate that the potential risk of increased ARV drug resistance with high levels of PrEP scale-up is offset by decreased incident infections, which alleviates this key concern [30,31]. Consequently, the appropriate response to emerging resistance signals is not restriction of prophylaxis programs but refinement of delivery models, including enhanced diagnostic algorithms to exclude acute infection, adherence reinforcement, and integration of resistance surveillance into routine monitoring frameworks. Our findings, based on a limited number of exposed individuals, underscore the need for larger studies to precisely quantify the risk-benefit balance, but they do not challenge the continued rollout of PrEP and PEP as effective HIV prevention strategies.

Analysis of resistance mutation patterns revealed that M184V, K103N, and M46L were the most common mutations associated with NRTI, NNRTI, and PI resistance, respectively. These mutations have been widely reported in both national and international studies and are known to compromise the efficacy of traditional first-line regimens, particularly NNRTI-based therapies [6]. The M184V and M184I mutations confer high-level resistance to both 3TC and FTC [26]. The K103N mutation is one of the most prevalent NNRTI mutations observed in China. It is a persistent intrahost mutation that confers reduced susceptibility to EFV and NVP [11,19,20]. Moreover, 0.4% (33/8798) of individuals carried PI resistance related to the M46L mutation, which conferred TDR to nelfinavir [11]. Currently, 3TC and EFV are widely used free drugs in first-line ART regimens in China [20]. Long-term use of a limited number of drugs facilitated the development and spread of drug resistance. Therefore, persistent surveillance of drug-resistant strains is required to accurately track and update the prevalence rates of major resistance genes in our region.

Despite the transition toward INSTI-based treatment and the emergence of long-acting ARV agents, continuous surveillance of traditional NRTI and NNRTI resistance remains highly relevant. First, NRTIs continue to serve as the backbone of most first-line regimens, and mutations such as M184V, M184I, and K65R may reduce the effectiveness of companion drugs even when combined with high-barrier INSTIs. Second, historical NRTI and NNRTI resistance profiles remain critical for constructing effective second-line and salvage regimens as archived resistance mutations can influence future treatment strategies. Third, because TDF and FTC constitute the pharmacological foundation of most PrEP programs worldwide, surveillance of resistance to these agents is essential for maintaining the effectiveness of biomedical HIV prevention strategies. Therefore, resistance monitoring should evolve from a single drug class approach to an integrated surveillance framework encompassing INSTI, capsid inhibitor, and traditional reverse-transcriptase inhibitor resistance.

This study has some limitations. First, the cross-sectional design precludes causal inference. Second, behavioral and prophylaxis-related information was self-reported and may be subject to recall and social desirability biases. Third, the small number of individuals with PrEP or PEP use and the low number of drug-specific resistance events limited the precision of our estimates. Although we used Firth logistic regression to reduce bias, the results for FTC and 3TC should be considered exploratory, and the TDF findings could not be adjusted for confounding. Fourth, we used conventional Sanger sequencing, which detects only major drug-resistant variants. However, as a recent study demonstrated, deep sequencing uncovers many low-abundance resistance mutations missed by conventional methods [7]; thus, our PDR prevalence was likely underestimated. Therefore, future studies in Zhejiang Province should adopt next-generation sequencing to capture minority variants and enhance early warning systems. Furthermore, resistance testing for INSTIs was not conducted as part of this investigation. Given the rising use of INSTIs in China, it becomes essential to investigate the impact of HIV-1 drug resistance on INSTIs.

In conclusion, Zhejiang Province currently exhibits an increasing burden of PDR within a genetically diverse HIV-1 epidemic. The association between CRF01_AE infection and elevated PDR risk underscores subtype-specific vulnerability. Although exposure prophylaxis was not linked to overall resistance, its association with FTC- and 3TC-specific resistance highlights the need for enhanced testing strategies and targeted resistance surveillance. Sustained integration of molecular epidemiology, biomedical prevention, and optimized ART selection will be essential to maintain the long-term effectiveness of HIV control efforts.

## Abbreviations

3TC: lamivudine
aOR: adjusted odds ratio
ART: antiretroviral therapy
ARV: antiretroviral
CDC: Center for Disease Control and Prevention
COMET: Context-Based Modeling for Expeditious Typing
EFV: efavirenz
EPV: events per variable
FTC: emtricitabine
INSTI: integrase strand transfer inhibitor
NNRTI: nonnucleoside reverse-transcriptase inhibitor
NRTI: nucleoside reverse-transcriptase inhibitor
NVP: nevirapine
OR: odds ratio
PCR: polymerase chain reaction
PDR: pretreatment drug resistance
PEP: postexposure prophylaxis
PI: protease inhibitor
PrEP: pre-exposure prophylaxis
RT-PCR: reverse-transcriptase polymerase chain reaction
TDF: tenofovir
TDR: transmitted drug resistance
